# Is there a role for an external beam boost in cervical cancer radiotherapy?^†^

**DOI:** 10.3389/fonc.2013.00003

**Published:** 2013-01-30

**Authors:** Rajni A. Sethi, Gabor Jozsef, David Grew, Ariel Marciscano, Ryan Pennell, Melissa Babcock, Allison McCarthy, John Curtin, Peter B. Schiff

**Affiliations:** ^1^Department of Radiation Oncology, New York University School of MedicineNew York, NY, USA; ^2^Department of Obstetrics and Gynecology, New York University School of MedicineNew York, NY, USA

**Keywords:** cervical cancer, brachytherapy, external beam radiotherapy

## Abstract

**Objectives:** Some patients are medically unfit for or averse to undergoing a brachytherapy boost as part of cervical cancer radiotherapy. In order to be able to definitively treat these patients, we assessed whether we could achieve a boost plan that would mimic our brachytherapy plans using external beam radiotherapy.

**Methods:** High dose rate brachytherapy plans of 20 patients with stage IIB cervical cancer treated with definitive chemoradiotherapy were included in this study. Patients had undergone computer tomography (CT) simulations with tandem and ovoids in place. Point “A” dose was 600–700 cGy. We attempted to replicate the boost dose distribution from brachytherapy plans using intensity-modulated radiotherapy (Varian Medical Systems, Palo Alto, CA, USA), volumetric modulated arc therapy (Rapid Arc, Varian Medical Systems, Palo Alto, CA, USA), or TomoTherapy (Accuray, Inc., Sunnyvale, CA, USA) with the brachytherapy 100% isodose line as our target. Target coverage, normal tissue dose, and brachytherapy point doses were compared with ANOVA. Two-sided *p*-values ≤0.05 were considered significant.

**Results:** External beam plans had excellent planning target volume (PTV) coverage, with no difference in mean PTV V95% among planning techniques (range 98–100%). External beam plans had lower bladder Dmax, small intestine Dmax, and vaginal mucosal point dose than brachytherapy plans, with no difference in bladder point dose, mean bladder dose, mean small intestine dose, or rectal dose. Femoral head dose, parametria point dose, and pelvic sidewall point dose were higher with external beam techniques than brachytherapy.

**Conclusions:** External beam plans had comparable target coverage and potential for improved sparing of most normal tissues compared to tandem and ovoid brachytherapy.

## INTRODUCTION

Localized cervical cancer (stage IB to IVA) is traditionally treated with concurrent chemoradiotherapy. Radiotherapy is usually administered through a three-dimensional conformal external beam approach to bring the whole pelvis to a minimum dose of 45 Gy, followed by a brachytherapy boost to give additional dose to the gross tumor within the cervix and parametria. This technique is often successful, with 5-year survival rates ranging from 60 to 70% (Rose et al., 1999; Pearcey et al., 2002; Eifel et al., 2004).

High dose rate (HDR) brachytherapy is commonly administered with a tandem and ovoid applicator. There are several benefits of tandem and ovoid brachytherapy. It allows delivery of a high radiation dose to the tumor site with rapid fall-off to protect normal tissue. In addition, organ motion is less concerning with an apparatus that is secured within target tissues of the pelvis. However, there are several downsides to the procedure. It is invasive, often requiring spinal or general anesthesia and conferring operative risks such as uterine perforation, infection, and bleeding. The tandem and ovoid apparatus is left in place for many hours and can be painful without strong sedative and narcotic medication.

Moderate to severe late complications of HDR brachytherapy are common. Although a recent meta-analysis reported the rates of moderate to severe late rectal or genitourinary toxicity to be 3% or less, some series have shown moderate or severe late toxicity rates of up to 23% for the rectum, 3% for the bladder, 6% for the small bowel, and 30% for the vagina (Clark et al., 1994; Pötter et al., 2000; Wang et al., 2010).

In light of the above risks, we questioned whether an external beam brachytherapy boost is achievable for patients undergoing definitive cervical cancer radiotherapy who are either medically unfit for or refuse a brachytherapy boost. Specifically, using external beam techniques, we tried to attain a similar dose distribution with comparable or improved normal-tissue sparing to that seen in previously treated brachytherapy plans at our institution.

Prior studies have evaluated the ability of external beam techniques to mimic brachytherapy plans; however, these studies included fewer patients and used only a single external beam technique (Wahab et al., 2004; Hsieh et al., 2010; Cengiz et al., 2012; Haas et al., 2012). We focused on a cohort of 20 patients and analyzed both volumetric and classical point dose endpoints to compare and contrast previously treated brachytherapy plans with three external beam techniques: volumetric modulated arc therapy (VMAT, Rapid Arc, Varian Medical Systems, Palo Alto, CA, USA), intensity-modulated radiotherapy (IMRT, Varian Medical Systems, Palo Alto, CA, USA), and TomoTherapy (Accuray, Inc., Sunnyvale, CA, USA).

## MATERIALS AND METHODS

This study was approved by the institutional review board. Twenty consecutive adult patients with a diagnosis of stage IIB cervical cancer treated with definitive chemoradiotherapy between 2003 and 2010 were included. All patients had received external beam radiotherapy to the whole pelvis followed by a tandem and ovoid HDR brachytherapy boost. Patients had undergone computer tomography (CT) simulation with tandem and ovoids in place prior to boost delivery. For each patient, we attempted to replicate the boost dose distribution from one of these planning CT scans using IMRT, VMAT, or TomoTherapy.

Doses of 600–700 cGy had been previously prescribed for each HDR brachytherapy fraction. The 100% prescription isodose volume from the tandem and ovoid procedure was used as the planning target volume (PTV) for external beam plans.

Normal tissues, including bladder, rectum, small intestine, and femoral heads, were contoured by the same physician (Rajni A. Sethi) on the planning CT scan and reviewed by a second physician (Peter B. Schiff). An IMRT plan using up to 11 non-coplanar beams was planned for 20 patients. A VMAT plan with two or three 270° arcs and a TomoTherapy plan were created for 10 of the study patients. **Table 1** presents the dose constraints used for the optimization of the external beam plans.

**Table 1:** Dose constraints used for intensity-modulated radiotherapy and volumetric arc radiotherapy plans.

Structure	Type	Volume (%)	Dose (cGy)
PTV	Upper	0	1000
PTV	Lower	100	710
Bladder	Upper	0	400
Bladder	Upper	3	300
Body	Upper	3	400
Femoral head	Upper	0	200
Rectum	Upper	0	300
Small intestine	Upper	0	200

The following dosimetric parameters were recorded: volume of PTV receiving 95% of the prescribed dose (V95), maximum dose to the PTV, and maximum and mean doses to the bladder, rectum, small intestine, and right and left femoral heads. In addition, point doses to brachytherapy points A, points B, points P, vaginal mucosal point, bladder point, and rectal point were also recorded.

The points were defined according to ICRU 38 (International Commission on Radiation Units and Measurements, 1985). The bladder point is defined on a lateral radiograph at the posterior surface of the balloon along an anteroposterior line drawn through the center of the Foley catheter balloon, and at the center of the balloon on an anterior/posterior (AP) radiograph. The rectal reference point is located, on a lateral radiograph, 5 mm behind the posterior vaginal wall at the level of the inferior end of the intrauterine. The pelvic sidewall reference point is located, on an AP radiograph, at the intersection of orthogonal lines drawn tangent to the highest and inner-most aspects of the acetabulum. On a lateral radiograph, it is at the level of the midpoint of a line drawn between the superior aspects of the right and left acetabula. Point A was defined on an AP radiograph 2 cm lateral to the plane of tandem and 2 cm superior to the cervical. Point B is located at the same level as Point A, 5 cm lateral from midline.

Because the prescribed dose for each brachytherapy plan ranged from 600 to 700 cGy, all dosimetric values were normalized to the prescription dose prior to statistical analysis and are presented as such in the results section. Single-factor ANOVA was used to test for differences in dose to the PTV, normal structures, or brachytherapy points among the four planning techniques. A two-tailed *t*-test was then used to test for differences between pairs of planning techniques for any parameters with significant differences on ANOVA. A two-sided *p*-value of 0.05 or less was considered significant for all statistical analyses.

## RESULTS

### DOSE TO TARGET VOLUME

**FIGURE 1:**
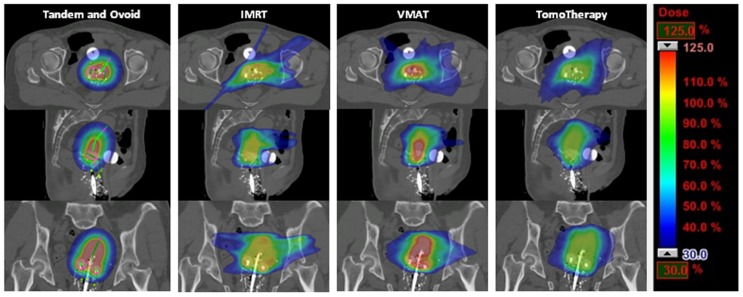
**Axial, sagittal, and coronal views of dose distribution among tandem and ovoid, intensity-modulated radiotherapy, volumetric modulated arc therapy, and TomoTherapy techniques for a representative patient**.

Dose distributions among the three planning techniques for a representative patient are shown in **Figure 1**. There was no difference in point A dose, either right or left, among the planning techniques, with a range of 97–102% of the prescription dose for all plans (*p* = 0.23 for left point A, and *p* = 0.20 for right point A, single-factor ANOVA). All plans had excellent PTV coverage when the PTV was defined as the treatment volume (within the 100% isodose curve) on the tandem and ovoid plan. Mean PTV V95 was 98% for IMRT and 100% for VMAT and TomoTherapy plans. The average maximum dose to the PTV was 112, 157, and 123% for IMRT, VMAT, and TomoTherapy plans, respectively (*p* < 0.001, two-tailed *t*-test).

### DOSE TO NORMAL STRUCTURES

Results of volumetric analyses of dose to normal tissues and analyses of brachytherapy point doses are presented in **Table 2**. **Table 3** shows the results of pair-wise comparisons of techniques for any of the parameters that were significantly different by ANOVA analysis.

**Table 2:** Comparison of dose to normal tissues and brachytherapy points, normalized to the prescription dose, among tandem and ovoid, intensity-modulated radiotherapy, and volumetric arc radiotherapy techniques.

	Mean (standard deviation)		
	IMRT	T&O	VMAT	Tomotherapy	*p*-value*
Rectum maximum dose	0.59 (0.14)	0.61 (0.25)	0.57 (0.09)	0.62 (0.11)	0.887
Rectum mean dose	0.20 (0.05)	0.23 (0.09)	0.19 (0.03)	0.23 (0.05)	0.280
Bladder maximum dose	0.98 (0.12)	1.47 (0.65)	1.15 (0.15)	1.03 (0.20)	**0.002**
Bladder mean dose	0.38 (0.09)	0.39 (0.09)	0.41 (0.06)	0.38 (0.05)	0.827
Small intestine maximum dose	1.00 (0.12)	1.30 (0.49)	1.01 (0.27)	1.01 (0.20)	**0.019**
Small intestine mean dose	0.22 (0.21)	0.21 (0.07)	0.15 (0.06)	0.19 (0.10)	0.553
Right femur maximum dose	0.31 (0.09)	0.12 (0.06)	0.35 (0.09)	0.34 (0.06)	**<0.001**
Right femur mean dose	0.08 (0.03)	0.05 (0.03)	0.12 (0.07)	0.15 (0.11)	**<0.001**
Left femur maximum dose	0.37 (0.10)	0.15 (0.07)	0.41 (0.10)	0.35 (0.09)	**<0.001**
Left femur mean dose	0.09 (0.03)	0.07 (0.05)	0.15 (0.07)	0.19 (0.11)	**<0.001**
Point A left	0.97 (0.03)	0.99 (0.06)	1.01 (0.08)	1.04 (0.07)	**0.020**
Point A right	0.98 (0.03)	0.98 (0.08)	1.02 (0.07)	1.05 (0.07)	**0.031**
Point B left	0.48 (0.13)	0.26 (0.04)	0.48 (0.13)	0.41 (0.04)	**<0.001**
Point B right	0.38 (0.09)	0.25 (0.05)	0.41 (0.08)	0.39 (0.04)	**<0.001**
Bladder point	0.56 (0.21)	0.62 (0.19)	0.57 (0.19)	0.54 (0.11)	0.727
Rectal point	0.49 (0.18)	0.47 (0.17)	0.49 (0.22)	0.51 (0.16)	0.938
Point P left	0.50 (0.14)	0.24 (0.07)	0.49 (0.13)	0.43 (0.06)	**<0.001**
Point P right	0.38 (0.13)	0.20 (0.06)	0.47 (0.13)	0.38 (0.07)	**<0.001**
Vaginal mucosal point left	1.03 (0.08)	1.44 (0.39)	0.99 (0.39)	1.08 (0.18)	**0.001**
Vaginal mucosal point right	1.03 (0.05)	1.37 (0.33)	0.99 (0.40)	1.07 (0.15)	**<0.001**

* Single-factor ANOVA.

*IMRT, intensity-modulated radiotherapy; T&O, tandem and ovoid; VMAT, volumetric arc radiotherapy.*

*Statistically significant ANOVA values are represented in bold.*

**Table 3:** Results of two-tailed *t*-tests for pair-wise comparisons of tandem and ovoid, intensity-modulated radiotherapy, volumetric modulated arc therapy, and TomoTherapy techniques for dosimetric parameters and doses to brachytherapy points that showed statistically significant differences on ANOVA analysis of normalized values.

	T&O vs. IMRT	T&O vs. VMAT	T&O vs. Tomo	IMRT vs. VMAT	IMRT vs. Tomo	VMAT vs. Tomo
Bladder Dmax	**0.001**	0.139	**0.044**	**0.002**	0.420	0.128
SI Dmax	**0.005**	0.100	0.031	0.899	0.933	0.974
R femur Dmax	**<0.001**	**<0.001**	**<0.001**	0.217	0.334	0.550
R femur mean	**0.037**	**0.002**	**0.001**	**0.078**	**0.011**	**0.027**
L femur Dmax	**<0.001**	**<0.001**	0.660	0.289	0.660	0.063
L femur mean	0.195	**0.001**	**0.001**	**0.002**	**0.002**	0.130
A left	0.293	0.432	**0.059**	0.051	**0.001**	0.441
A right	0.796	0.228	**0.039**	**0.024**	**0.001**	0.467
B left	**<0.001**	**<0.001**	**<0.001**	0.980	0.137	0.092
B right	**<0.001**	**<0.001**	**<0.001**	0.512	0.877	0.271
P left	**<0.001**	**<0.001**	**<0.001**	0.828	0.126	0.301
P right	**<0.001**	**<0.001**	**<0.001**	0.116	0.894	0.084
Mucosa right	**<0.001**	**0.019**	**0.007**	0.698	0.340	0.313
Mucosa left	**<0.001**	**0.011**	**0.021**	0.730	0.344	0.996

*T&O, tandem and ovoid; IMRT, intensity-modulated radiotherapy; VMAT, volumetric arc radiotherapy; Tomo, TomoTherapy. Statistically significant ANOVA values are represented in bold.*

Target coverage was excellent with all three external beam plans. There was no difference in average PTV-V95% among the three external beam techniques: 98% for IMRT, 100% for VMAT, and 100% for tandem and ovoid plans (*p* = 0.122, single-factor ANOVA). PTV Dmax was significantly higher for VMAT compared to IMRT or TomoTherapy, averaging 157, 112, and 123%, respectively (*p* < 0.001, single-factor ANOVA).

Tomotherapy had higher dose to point A than other planning techniques. All three external beam techniques had higher point B and point P doses than tandem and ovoid plans.

The maximum dose to the bladder was lower with external beam plans compared to brachytherapy plans. IMRT and TomoTherapy achieved the lowest bladder Dmax. There was no difference in bladder mean dose or dose to the bladder point among planning techniques.

Rectal point dose, maximum dose to the rectum, and mean rectal dose were not different among the planning techniques.

Small intestine maximum dose was lower with external beam plans compared to brachytherapy plans, although no difference in mean dose was found.

Vaginal mucosal point doses were lower with external beam techniques compared to tandem and ovoid plans.

Femoral head mean and maximum doses were lower with brachytherapy than with external beam techniques, with the exception of left femur mean dose which was not on average different between IMRT and tandem and ovoid plans. Of the external beam techniques, TomoTherapy incurred the highest femur mean doses, and IMRT incurred the lowest femur mean doses.

## DISCUSSION

We were able to obtain good coverage of the volume treated by brachytherapy with improved normal tissue sparing with all three external beam techniques. The most common sites of late complications from tandem and ovoid brachytherapy are, in order of decreasing incidence, vagina, rectum, small intestine, and bladder (Pötter et al., 2000; Chen et al., 2003; Wang et al., 2010). In this study, IMRT, VMAT, and TomoTherapy enabled a reduction in vaginal mucosal dose as well as maximum dose to the bladder and small intestine. IMRT and TomoTherapy were better able to spare the bladder than VMAT, and TomoTherapy had the highest femoral head doses. The external beam plans were otherwise very similar. Because maximum dose to pelvic organs has been correlated with late complication rates, use of an external beam technique could theoretically result in fewer late tissue effects (Petereit and Pearcey, 1999).

The dosimetric differences between the external beam plans may be decreased or their quality improved by using different optimization criteria for each modality. In this study we used the same optimization criteria for a simpler comparison.

We used the prescription isodose curve from the tandem and ovoid plan to define our PTV; however, an alternative method of target definition would be required for clinical implementation of an external beam boost, since tandem and ovoids will not be in place. We anticipate using magnetic resonance imaging (MRI) to assist with tumor and target definition, as several groups have successfully done in clinical trials of image-guided brachytherapy for locally advanced or recurrent cervical cancers (Fokdal et al., 2011; Pötter et al., 2011).

Another consideration in planning an external beam boost is the substantial organ motion and deformation displayed by the pelvic organs, especially, the bladder, rectum, and uterus (Chan et al., 2008; Lim et al., 2011). With brachytherapy, this motion is overcome because the applicator is fixed within the target tissue. With an external beam technique, target motion becomes an important concern, and internal target volume margins would need to be defined to allow for both organ motion and deformation during and between treatments. It is possible that, with the addition of an internal target volume (ITV) expansion, dose to organs at risk may be higher than what is represented here. However, with MRI-based planning to identify a gross tumor volume (GTV), final PTV volume, and corresponding dose to organs at risk, may actually be lower than what is presented here. Finally, image guidance and/or use of fiducial markers would be required to account for inter-fraction motion.

A characteristic of brachytherapy is the rapid dose fall-off with distance from the source. This means that, while the prescription point or volume receives the prescription dose, high risk tissues near the source receive a substantially higher dose. In this study, target coverage with the prescription dose was excellent with external beam techniques; however, external beam techniques do not capture the substantial dose inhomogeneity seen with brachytherapy. We used a constraint to limit the maximum dose in external beam plans, due to the uncertainty of hotspot location, which could adversely impact a critical structure. The question then arises as to the biological and clinical significance of this effect. There has been no correlation between HDR fraction schedule or biologic equivalent dose with pelvic control or overall survival, for many common fractionation regimens (Petereit and Pearcey, 1999). Also, there has been no proven benefit of high dose rate brachytherapy over low dose rate brachytherapy in terms of pelvic control or survival (Wang et al., 2010). However, presently it is uncertain which dosimetric parameter, such as minimum, average, or maximum tumor dose, is associated with tumor control. Therefore, it is unclear whether our external beam techniques, which maintained minimum tumor dose but had a lower maximum dose, is biologically equivalent to brachytherapy. Clinical implementation of an external beam boost should accordingly be done in the setting of a prospective clinical trial.

External beam techniques will expose more normal tissue to low radiation doses through beam entry and exit paths, as evidenced by the higher femoral head doses in external beam plans in this study. Increased volume of tissue with low dose exposure may incur a higher risk of second malignancy, which could be a potential drawback of an external beam approach (Hall and Wuu, 2003; Purdy, 2008). This issue is likely to be of minimal importance in patients already receiving external beam radiation.

The intended application of this technique is not to replace brachytherapy as a modality, but rather as a tool for situations when brachytherapy is not available. For example, patients who are medically unfit for or refuse a brachytherapy procedure may receive definitive treatment of their cancer. Radiation oncology practices without brachytherapy expertise or equipment could definitively treat their patients, which could particularly impact patients in medically underserved areas. Although we mimic an HDR brachytherapy dose schedule in this study, alternative fractionation regimens, such as a daily concomitant boost or stereotactic body radiotherapy, could be used within a research setting to decrease total treatment time. This potentially could lead to improved tumor control given prior evidence that overly prolonged treatment schedules can negatively impact outcomes (Lanciano et al., 1993; Chen et al., 2003).

In summary, external beam boost plans had good target coverage with the prescription dose and had improved normal tissue sparing compared to brachytherapy plans. Due to important considerations such as target motion and target delineation, this technique is limited in scope compared to traditional brachytherapy and should be clinically implemented in the setting of a prospective feasibility trial.

## Conflict of Interest Statement

The authors declare that the research was conducted in the absence of any commercial or financial relationships that could be construed as a potential conflict of interest.
